# Reproductive Health and Assisted Conception in Celiac Disease and Non-Celiac Gluten Sensitivity: A Narrative Review

**DOI:** 10.3390/nu17132215

**Published:** 2025-07-03

**Authors:** Efthalia Moustakli, Panagiotis Christopoulos, Anastasios Potiris, Athanasios Zikopoulos, Eirini Drakaki, Theodoros Karampitsakos, Ismini Anagnostaki, Nikolaos Kathopoulis, Periklis Katopodis, Apostolia Galani, Chrysi Christodoulaki, Athanasios Zachariou, Peter Drakakis, Sofoklis Stavros

**Affiliations:** 1 Laboratory of Medical Genetics, Faculty of Medicine, School of Health Sciences, University of Ioannina, 45110 Ioannina, Greece; ef.moustakli@uoi.gr (E.M.); katopodisper@gmail.com (P.K.); 2 Second Department of Obstetrics and Gynecology, Aretaieion University Hospital, Medical School, National and Kapodistrian University of Athens, 11528 Athens, Greece; panchrist@med.uoa.gr; 3 Third Department of Obstetrics and Gynecology, University General Hospital “ATTIKON”, Medical School, National and Kapodistrian University of Athens, 12462 Athens, Greece; thanzik92@gmail.com (A.Z.); theokarampitsakos@hotmail.com (T.K.); isanagnostaki3@gmail.com (I.A.); christodoulakichr@hotmail.com (C.C.); pdrakakis@med.uoa.gr (P.D.); sfstavrou@med.uoa.gr (S.S.); 4 First Department of Obstetrics and Gynecology, Alexandra Hospital, Medical School, National and Kapodistrian University of Athens, 11528 Athens, Greece; eirinidrak@med.uoa.gr (E.D.); nickatho@med.uoa.gr (N.K.); 5 Department of Metabolism, Digestion and Reproduction, Faculty of Medicine, Imperial College London, London W12 ONN, UK; l.galani22@imperial.ac.uk; 6 Department of Urology, School of Medicine, Ioannina University, 45110 Ioannina, Greece; zahariou@otenet.gr

**Keywords:** gluten, diet, infertility, assisted reproductive technologies (ARTs), non-celiac gluten sensitivity (NCGS)

## Abstract

The increasing use of assisted reproductive technologies (ARTs) globally, such as intracytoplasmic sperm injection (ICSI) and in vitro fertilization (IVF), has highlighted the pressing need to determine the modifiable factors affecting the success of implantation and the outcomes of early pregnancy. Scientific interest in the role of nutrition in fertility is growing, but outside of celiac disease, little is known about gluten, a dietary protein with immunogenic and inflammatory properties. With an emphasis on ART results, this narrative review summarizes the most recent data regarding the possible effects of gluten consumption on reproductive health, focusing primarily on individuals with celiac disease (CD) and non-celiac gluten sensitivity (NCGS). In addition to discussing potential molecular processes connecting gluten-induced inflammation, increased gut permeability, autoimmune, and decreased endometrial receptivity, we further explore the documented link between CD and infertility and investigate new information on NCGS. These findings are tentative and based on scant low-quality evidence, although some case reports and small clinical studies have indicated that avoiding gluten may help some people undergoing ART, especially those with immune-mediated diseases or infertility that cannot be explained. There is currently no robust prospective evidence confirming that gluten restriction improves infertility outcomes. Therefore, before gluten elimination is advised in this situation, more carefully planned extensive research is required to generate reliable scientific proof. Beyond traditional celiac disease, we suggest that gluten sensitivity might be an underappreciated factor in ART failure that merits more research. A gluten-free diet may serve as a low-risk supplementary option for appropriately selected patients, pending the results of more extensive controlled studies.

## 1. Introduction

The growing use of ART, including IVF and ICSI, has drastically changed the family-building environment globally. As the use of these therapies increases, there is a growing need to identify modifiable factors that can influence implantation success and early pregnancy outcomes. There has been a rising focus on the role of nutrition in fertility and reproductive health. Despite extensive research on various dietary components, the specific effects of gluten, a protein complex found in wheat, barley, rye, and triticale, are still not clearly defined [1].

More than 1% of people worldwide suffer from celiac disease (CD), an autoimmune condition that is particularly common. Beyond CD, non-celiac gluten sensitivity (NCGS) may affect between 0.5% and 6% of the population, although estimates vary depending on diagnostic criteria. Notably, higher prevalence figures (e.g., up to 13%) have been reported in selected patient populations such as those with lymphocytic duodenosis [2]. These figures should therefore be interpreted with caution and are not representative of the general population. For a systematic analysis of prevalence, please refer to Molina-Infante et al. (2015) [3]. In vulnerable individuals, gluten can trigger inflammatory and immunological reactions. Its intake leads to systemic inflammation, impaired nutrient absorption, and villous atrophy in patients with CD. These conditions have been associated with reproductive challenges such as infertility, recurrent miscarriages, and negative perinatal outcomes [4,5].

Crucially, gluten-related diseases are not limited to CD. The hallmark of NCGS is gluten-induced gastrointestinal and extraintestinal symptoms; it lacks the autoimmune characteristics of CD and the IgE-mediated profile of wheat allergy. Although it is still poorly understood, the prevalence and effects of NCGS on reproductive function, especially on ART, represent a developing clinical interest [6].

This narrative review aims to review key research and recent evidence concerning gluten-related conditions, specifically CD and NCGS, and their possible influence on ART outcomes and reproductive health. Before evaluating early research on NCGS and its reproductive implications, we examine the known connection between CD and subfertility. Due to the significant differences in the underlying mechanisms and clinical implications, studies involving healthy persons who do not have gluten-related illnesses are typically excluded. Highlighting possible biological pathways, highlighting current knowledge gaps, and providing a targeted overview are the objectives. Scientific rigor is crucial, even though narrative reviews do not adhere to the strict guidelines of systematic reviews. As stated, this review was conducted following accepted guidelines for the narrative literature reviews, which include specific goals, methodical literature searches, and an organized synthesis of findings [7,8]. We aimed to present a fair and fact-based summary of the recent conclusions in CD, NCGS, and reproductive health.

Eliminating gluten may help some patient populations undergoing ART, particularly those with immunologic malfunction or infertility that cannot be explained, according to limited clinical data, including case reports and small observational studies.

We further highlight that patients adhering to a gluten-free diet (GFD) may still experience nutritional deficiencies commonly seen in CD, such as iron, folate, and vitamin D deficiencies, which may independently affect reproductive outcomes. While gluten sensitivity is a potentially modifiable factor influencing ART outcomes, it is important to consider other contributing factors including hormonal imbalances, autoimmune conditions, and lifestyle variables. Additionally, eliminating gluten through a GFD may inadvertently lead to nutritional deficiencies that could negatively impact reproductive health, underscoring the need for careful dietary monitoring and management when adopting such restrictive diets.

## 2. Methodology

The purpose of this narrative review was to compile the most recent data about the effects of CD and NCGS on the outcomes of assisted conception and reproductive health. According to Ferrari (2015) and Sukhera (2022), this review was conducted using recognized methodological guidelines for narrative literature reviews to increase transparency and reduce selection bias [7,8].

### 2.1. Literature Search Strategy

A comprehensive literature search was performed using three major databases: PubMed/MEDLINE, Scopus, and Web of Science. The search encompassed research articles released between January 2000 and December 2024. “Celiac disease”, “non-celiac gluten sensitivity”, “gluten”, “infertility”, “reproductive health”, “pregnancy outcomes”, “assisted reproductive technologies”, “IVF”, “ICSI”, “implantation failure”, and “recurrent miscarriage” were among the keywords and MeSH terms that were used both singly and in combination. Only English-language publications and research involving human participants were included by applying filters.

### 2.2. Study Selection and Data Synthesis

Studies that assessed ART outcomes or reproductive health outcomes in patients with CD or NCGS diagnoses were deemed eligible. Clinical trials, systematic reviews, meta-analyses, observational studies, and pertinent narrative reviews were among the study designs that qualified. When they offered clinically significant insights, case reports and expert commentary were also taken into consideration in regions with little evidence, especially for NCGS. Non-English publications, research unrelated to reproductive health outcomes, and animal studies were among the exclusion criteria.

Two authors independently screened titles and abstracts for relevance. Potentially qualifying articles’ full texts were examined before being included. Discussions with a third author helped to settle disagreements on research selection. Due to the narrative character of this study, the results were qualitatively synthesized, emphasizing clinical implications, biological mechanisms, and knowledge gaps.

## 3. Gluten-Related Disorders and Their Systematic Effects

Gluten-related disorders include a range of immune and non-immune illnesses triggered by gluten consumption. CD is the most extensively studied gluten-related disorder. It occurs in genetically predisposed individuals and triggers an inflammatory reaction to gluten that damages the small intestine. Malabsorption and systemic inflammation resulting from CD can negatively impact multiple physiological systems, including reproductive function [9,10].

CD frequently disrupts the absorption of vital micronutrients like iron, folate, vitamin B12, calcium, and vitamin D, crucial for ovulation, implantation, and fetal development. For instance, iron deficiency anemia has been linked to poor outcomes for both the mother and the fetus, and folate is essential for neural tube development [11]. Type 1 diabetes and Hashimoto’s thyroiditis are two other autoimmune conditions that frequently coexist with CD and can further reduce fertility [12].

Unexpected infertility, delayed menarche, amenorrhea, recurrent miscarriages, intrauterine growth restriction (IUGR), premature birth, and low birth weight have all been linked to women with untreated CD [13]. Crucially, by enhancing nutrient absorption, lowering systemic inflammation, and reestablishing gut health, stringent GFD adherence can undo many of these consequences [14]. In some cases, fertility improves without ART intervention in individuals with CD or NCGS, and, in patients undergoing ART within these populations, GFD adherence has been linked to improved implantation and pregnancy outcomes [15,16].

Though CD is the most clearly defined gluten-related disorder, increasing attention is being paid to NCGS and wheat allergy. NCGS is believed to involve immune activation without the characteristic intestinal damage seen in CD and shares several extraintestinal symptoms with it. Because of the reported symptom overlap and potential common mechanisms, its impact on reproductive health is increasingly recognized [17].

**Figure 1:**
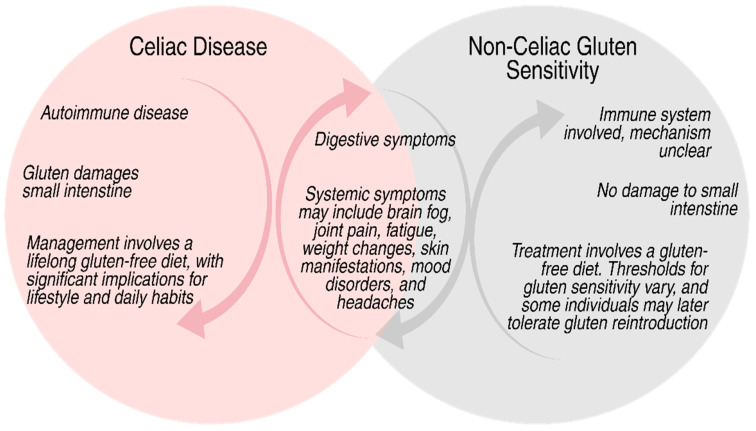
This Venn diagram illustrates the similarities and differences between CD and NCGS. While both can cause digestive and systemic symptoms, CD is an autoimmune condition that damages the small intestine and requires a lifelong gluten-free diet. NCGS involves the immune system without intestinal damage, and some individuals may eventually tolerate gluten.

Gluten-related disorders, particularly celiac disease, may impair reproductive health through inflammatory responses, chronic inflammation, and nutritional deficiencies. Optimizing reproductive outcomes requires acknowledging and addressing these consequences, which may serve as the foundation for tailored nutritional interventions in certain patient populations [18]. Figure 1 illustrates the similarities and differences between CD and NCGS.

## 4. Impact of Gluten-Related Disorders on Female Reproductive Health

A variety of gynecological and reproductive conditions in women, especially those who are experiencing infertility that cannot be explained, have long been linked to CD. According to studies, the prevalence of CD is much higher in women who experience infertility that cannot be explained than in the general population [19]. Estimates vary from 2% to 8%, depending on the cohort and diagnostic criteria. This overrepresentation raises the possibility that immunological dysregulation linked to gluten-related disorders, particularly in patients with celiac disease (CD), may be a significant and under-recognized factor in female reproductive failure [20].

One of the earliest reproductive manifestations of undiagnosed CD is menstrual irregularity, including delayed menarche, oligomenorrhea, and secondary amenorrhea. The systemic inflammation and persistent nutritional deficiencies that define untreated CD are commonly associated with these abnormalities. A lack of essential micronutrients like iron, folate, and vitamin D can worsen disruptions in hypothalamic–pituitary–ovarian signaling, leading to impaired ovarian function [21,22]. Although further research is needed to confirm this connection, there is mounting evidence that untreated CD may reduce ovarian reserve, as measured by anti-Müllerian hormone (AMH) and antral follicle counts [23]. In CD, circulating autoantibodies, cytokine profile alterations, and local inflammation may decrease endometrial receptivity, which could impede early placentation and implantation [24]. Untreated celiac disease has also been associated in studies with a higher risk of IUGR, low birth weight, premature birth, and repeated miscarriages [25]. These outcomes may stem from a combination of poor maternal nutrition, autoimmune dysfunction, and impaired placental development, although a direct causal relationship has not been established [26].

It is noteworthy that several of these reproductive abnormalities have been demonstrated to be reversed by rigorous adherence to a GFD. Initiating a GFD has improved ovulatory function, restored regular menstrual cycles, and raised the likelihood of conception in women with CD, sometimes without the need for ART [27]. Due to increased nutrient absorption and decreased systemic inflammation, women with CD who follow a GFD in ART settings appear to have greater implantation and pregnancy rates than their untreated counterparts, according to a limited observational study and case series [28].

Although little is known about how NCGS affects the reproductive health of women, suspicions are rising. According to some accounts, women who test negative for CD or wheat allergy but have endometriosis-like symptoms, repeated miscarriages, or infertility that cannot be explained respond well to gluten exclusion. Immune activation, intestinal permeability, and mild nutrient malabsorption in NCGS are thought to be factors in subfertility in some patients, despite the absence of mechanistic research [29,30]. A comparative overview of gluten’s reproductive effects in males and females is summarized in Table 1.

## 5. Impact of Gluten-Related Disorders on Male Reproductive Health

Although the majority of research on gluten-related disorders and fertility has predominantly focused on female populations, emerging evidence indicates that celiac disease and associated gluten sensitivity may have detrimental effects on male reproductive health. The current research indicates a potential association between untreated celiac disease in men and both decreased semen quality and hormonal abnormalities [18,22] (Table 1).

Studies indicate that men with celiac disease may exhibit abnormal semen characteristics, including atypical sperm morphology, reduced motility, and lower sperm count. Zinc and selenium are especially important for spermatogenesis and antioxidant defense within the seminal plasma [39].

Untreated CD has been associated with hypogonadism and disrupted regulation of the hypothalamic–pituitary–gonadal (HPG) axis, potentially due to low body mass index, chronic illness, and micronutrient deficiencies [40,41]. Low testosterone levels accompanied by increased FSH and LH may indicate primary testicular dysfunction in some individuals. These disturbances can potentially improve with nutritional restoration following the adoption of a GFD [42].

Men with CD may find it more difficult to conceive if they also have coexisting autoimmune diseases such as type 1 diabetes or autoimmune thyroiditis [43]. Although conclusive evidence is lacking, there is a theoretical concern that gluten intake may impair sperm function through the development of anti-sperm antibodies or cross-reactive autoimmune mechanisms [26].

It is heartening to learn that research indicates that, while following a GFD, men with CD can experience measurable improvements in their sperm quality, hormone profiles, and overall reproductive capacity [44]. Given other indicative symptoms or a family history of gluten sensitivity, these results lend credence to the idea that gluten exclusion may provide a low-risk non-invasive treatment for a subset of male patients with infertility that cannot be explained or abnormal semen tests [45].

There is currently very little information available on NCGS in male fertility, and more investigation is required to ascertain whether immune responses to gluten that are not celiac disease may directly impact male reproductive function [45].

## 6. Gluten-Related Disorders and Assisted Conception Outcomes

ART, including IVF, ICSI, and IUI, has become a vital therapy option for infertile individuals and couples. Despite significant progress, the success rates of assisted reproductive technologies remain markedly variable and are determined by numerous factors, several of which may be amenable to intervention. Recent studies suggest that gluten-related disorders may represent a significant but often overlooked factor affecting the efficacy of ART [46,47].

It is commonly known that untreated CD can lead to infertility issues and unfavorable pregnancy outcomes. Oocyte quality, assessed through parameters such as meiotic spindle integrity, mitochondrial function, chromosomal alignment, and morphological features, may be negatively impacted by the inflammatory mechanisms, persistent intestinal inflammation, and nutrient malabsorption characteristic of CD [48]. Deficiencies in antioxidants, folate, and vitamin B12, which are frequently observed in individuals with celiac disease, have been demonstrated to impair mitochondrial function and increase oxidative stress in oocytes, potentially diminishing their developmental competence [41]. These alterations, in combination with pro-inflammatory cytokine activity, have the potential to adversely affect embryonic development and impair endometrial receptivity, thereby substantially lowering the probability of successful implantation and pregnancy [25]. According to a study showing improved ART outcomes in patients with diagnosed CD who follow a strict GFD, removing gluten from the diet may provide a more favorable reproductive environment. The observed association, in certain instances, between commencing a gluten-free diet prior to ART and improved implantation and live birth rates has underscored the potential role of gluten-mediated systemic effects in influencing ART success [14].

There is little clarity regarding NCGS’s effect on ART. Similar to CD, NCGS may cause systemic inflammation and increased intestinal permeability, but little is known about how it affects fertility and the results of ART. Limited case series and anecdotal reports indicate that gluten consumption may negatively impact sensitive individuals undergoing ART; however, more comprehensive and rigorous research is needed to validate these observations [29,45].

In terms of mechanism, ART depends on the quality of the gametes and the receptivity of the uterine lining. Chronic inflammation and immune responses triggered by gluten may negatively affect the endometrial environment or cause immune attacks on the embryo, which could hinder implantation. Further restricting the success of ART are vitamin deficiencies, which are common in gluten-related illnesses and can affect sperm parameters and folliculogenesis. These molecular mechanisms help explain the clinical observations linking gluten-related issues to reproductive challenges during assisted conception [24,49].

Ultimately, the evidence connecting gluten to ART outcomes is promising yet still preliminary. Further comprehensive research is necessary to clarify these connections and inform therapeutic approaches. In the meantime, increasing awareness of gluten-related factors in patients facing infertility or repeated ART failure may lead to personalized nutritional interventions and better reproductive outcomes [27,50].

## 7. Biological Mechanisms in Gluten-Related Reproductive Dysfunction

Clinical associations between gluten-related disorders and reproductive abnormalities are supported by a number of overlapping biological pathways. A detailed understanding of these mechanisms is vital for accurately assessing the impact of gluten consumption on reproductive outcomes and for informing the design of advanced therapeutic approaches [29,51]. A summary of these mechanisms and their reproductive impacts is presented in Table 2.

### 7.1. Immune Activation and Autoimmunity in Affected Individuals

The maladaptive immune response to gluten peptides, which underpins CD, activates both the innate and adaptive immune systems. This immune activation results in chronic intestinal inflammation and the production of systemically circulating autoantibodies, including anti-tissue transglutaminase [52]. These pro-inflammatory mediators and autoantibodies may directly attack reproductive organs or disrupt the intricate process of embryo implantation. Thyroiditis and other autoimmune comorbidities are common in patients with CD and independently contribute to infertility and pregnancy difficulties, despite the lack of conclusive evidence for reproductive tissue autoimmunity in CD [24].

### 7.2. Chronic Inflammation and Cytokine Dysregulation in Gluten-Related Conditions

Gluten ingestion has been shown to trigger an increase in pro-inflammatory cytokines, such as interleukin-6 (IL-6), interferon-gamma (IFN-γ), and tumor necrosis factor-alpha (TNF-α), in susceptible individuals, contributing to systemic immune activation. These cytokines, which have been well-documented to inhibit ovarian follicle development, disrupt HPG axis signaling, and establish an unfavorable uterine milieu, have a deleterious effect on implantation and the early stages of placental development [56]. Men’s testicular tissue can be impaired, and spermatogenesis reduced by chronic inflammation. Therefore, one important mediator of reproductive failure may be the systemic inflammation caused by gluten [57].

### 7.3. Increased Intestinal Permeability “Leaky Gut” in Gluten-Sensitive Patients

Increased intestinal permeability is a hallmark of celiac disease and potentially NCGS. Gluten peptides have been shown to compromise the integrity of tight junction proteins, resulting in increased intestinal permeability, commonly described as “leaky gut”, which facilitates the translocation of luminal antigens and microbial products into the systemic circulation. Sustained inflammation and systemic immune activation arising from this process can impact reproductive tissues as well as other distant organs. Consequently, this immune dysregulation may lead to reduced endometrial receptivity and impaired gamete function [58,59].

### 7.4. Nutrient Malabsorption in Individuals with Gluten-Related Disorders

Villous atrophy associated with celiac disease leads to malabsorption of vital micronutrients essential for reproductive function. Common deficiencies in iron, folate, vitamin B12, vitamin D, calcium, zinc, and selenium are closely associated with poor gametogenesis, hormonal disruptions, and negative pregnancy outcomes [60,61]. For instance, iron deficiency anemia reduces oxygen delivery to both maternal and fetal organs, whereas folate deficiency increases the risk of neural tube defects and miscarriage. Although strict adherence to a GFD is maintained, micronutrient deficiencies can persist, making it essential to apply targeted nutritional interventions during the preconception and perinatal periods to support positive reproductive and developmental outcomes [62,63].

### 7.5. Hormonal Imbalances Related to Gluten-Related Conditions

Micronutrient deficiencies and chronic inflammatory conditions commonly associated with gluten-related disorders may disrupt the delicate hormonal balance maintained by the HPG axis. Women may encounter abnormalities in the luteal phase or anovulation, while men may suffer from hypogonadism and decreased testosterone synthesis. These hormonal shifts lower reproductive potential and degrade gamete quality [64,65].

### 7.6. Oxidative Stress in Gluten-Affected Individuals

Oxidative stress is a recognized contributor to male infertility, and inflammation induced by gluten exposure may exacerbate the levels of reactive oxygen species (ROS) in seminal plasma. Deficiencies in antioxidant micronutrients such as zinc and selenium further compromise the body’s ability to neutralize ROS, potentially leading to sperm DNA damage, decreased motility, and impaired fertilization. For males with CD-related infertility, addressing oxidative stress with diet and supplements may be very important [66].

### 7.7. Emerging Mechanisms: Epigenetics and Microbiota in Gluten-Related

Recent studies have demonstrated that gluten may indirectly impact reproductive health by modulating the gut microbiota and epigenetic mechanisms. Gluten sensitivity and the dysbiosis observed in CD may alter hormone metabolism and systemic immune responses. Furthermore, epigenetic modifications induced by chronic inflammation may affect gene expression related to fertility and embryonic development, although these hypotheses require further investigation [67,68].

Significant effects on reproductive health outcomes are caused by the gut microbiota’s modulation of the intricate relationships between diet, immunological response, and hormone control. Prolonged consumption of gluten or adherence to a GFD significantly impacts the gut microbiota, leading to decreased microbial diversity and reduced levels of beneficial bacteria such as Lactobacillus and Bifidobacterium [69]. These alterations may jeopardize the synthesis of short-chain fatty acids (SCFAs), which are crucial metabolites that regulate immunological responses and preserve the integrity of the intestinal barrier. Dysbiosis can disrupt the estrobolome, the community of gut microbes responsible for estrogen metabolism, thereby influencing endometrial receptivity, the hypothalamic pituitary gonadal axis, and systemic hormone levels [70,71].

Furthermore, dysbiosis-associated increased intestinal permeability, commonly referred to as ‘leaky gut’, facilitates the translocation of bacterial components into systemic circulation, triggering inflammation that adversely affects ovarian function, implantation, and the maintenance of pregnancy [69]. Micronutrient deficiencies essential for fertility are aggravated by malabsorption linked to alterations in the gut microbiota. Finally, alterations in the gut microbiota may induce local and systemic disruptions in immune regulation, potentially leading to immune-mediated infertility and reduced success rates in ART. Although clinical evidence is currently scarce, these molecular findings imply that microbiota-directed therapeutic strategies may offer potential benefits for reproductive outcomes in gluten-related disorders [72].

## 8. Discussion

Emerging evidence suggests that gluten may indirectly affect reproductive health by influencing gut microbiota composition and epigenetic regulatory processes. Gluten sensitivity and the gut dysbiosis associated with celiac disease have the potential to affect hormone metabolism and systemic immune responses [73]. Moreover, epigenetic modifications driven by chronic inflammation may influence the expression of genes associated with fertility and embryonic development; however, these assertions require additional empirical validation. Professional bodies such as the American College of Obstetricians and Gynecologists (ACOG) and the European Society of Human Reproduction and Embryology (ESHRE) recommend CD screening for women with specific gastrointestinal complaints, unexplained infertility, or recurrent pregnancy loss [74]. Nonetheless, given the lack of standardized diagnostic guidelines and insufficient data on the effects of NCGS on fertility, routine screening for NCGS in reproductive-age individuals or patients undergoing assisted reproduction is not presently advised [51].

To address persisting micronutrient deficits despite a gluten-free diet, nutritional counseling is highly recommended for women with CD or gluten-related diseases, especially during preconception and pregnancy. To improve reproductive and pregnancy outcomes, iron, folate, vitamin D, calcium, and other vital minerals are frequently required as supplements [75].

Patients with CD must strictly follow a GFD to improve their reproductive prospects. Nonetheless, objectively assessing adherence remains challenging, especially in contexts such as ART and infertility, where even minimal gluten exposure may have detrimental effects. Conventional assessment methods relying on dietitian evaluation or patient self-report often lack sufficient accuracy and reliability [76].

As a promising non-invasive biomarker of recent gluten ingestion, recent developments have made it possible to detect gluten immunogenic peptides (GIPs) in urine or feces [77,78]. These peptides enable more precise monitoring of dietary compliance by providing an objective measure of gluten intake. The personalized treatment of reproductive dysfunction linked to gluten intake may become feasible through the integration of GIP monitoring into clinical practice and research methodologies. This approach represents an effective strategy to optimize reproductive outcomes and reduce ART failures associated with inadvertent gluten exposure [79].

More people are becoming aware of the possible advantages of avoiding gluten for patients undergoing ART, particularly those with CD or infertility that cannot be explained. The existing evidence remains preliminary, and no established guidelines currently recommend gluten-free diets as a routine adjunct to ART treatment. To properly adjust dietary treatment regimens and incorporate the diagnosis of gluten-related diseases into the overall infertility assessment, clinicians should take a patient-specific approach [21].

Ultimately, although the research on the influence of gluten on ART and reproductive health is still emerging, current recommendations underscore the critical importance of identifying and treating celiac disease to optimize fertility outcomes [27]. Comprehensive research is warranted to formulate evidence-based guidelines regarding gluten avoidance in non-celiac populations and to facilitate the integration of these protocols within reproductive medicine frameworks [80].

Although still in its early stages, research on the impact of gluten and gluten-related disorders on assisted conception and reproductive health is rapidly advancing. Beyond CD, recent findings indicate that conditions such as NCGS may impact fertility and ART outcomes by inducing immune system activation, systemic inflammation, and nutritional deficiencies [81,82]. Although comprehensive data remain scarce, existing case reports and small observational studies propose that gluten exclusion may be advantageous for certain patient groups, especially those presenting with immune-mediated reproductive disorders or idiopathic infertility [27].

A key research objective for the future is to carry out large-scale rigorously designed clinical trials to determine the effects of gluten-free diets on ART success rates and pregnancy outcomes in both celiac and non-celiac individuals [81,83]. Exploring biomarkers of gluten sensitivity associated with reproductive dysfunction may lead to improved screening and diagnosis, paving the way for more tailored treatment strategies. Clinical interventions can be more effectively guided if the molecular pathways linking gluten intake to reproductive dysfunction are better understood [84].

Understanding how the gut microbiota influences the effects of gluten on fertility is crucial, given that gut dysbiosis may play a role in hormonal disturbances and systemic inflammation. To promote the health of both mother and fetus, it will be essential to conduct nutritional research aimed at identifying the best micronutrient management strategies for gluten-related conditions during preconception and pregnancy [55,85].

Incorporating gluten-related tests into standard fertility tests may prove to be a beneficial part of reproductive care, particularly in situations of infertility that cannot be explained or repeated ART failure. Multidisciplinary collaboration between immunologists, gastroenterologists, dietitians, and reproductive specialists will be crucial to advancing research and applying new findings in clinical practice [21,22].

Patients experiencing persistent gastrointestinal symptoms or infertility who initiate a GFD prior to formal diagnostic evaluation present an added challenge to accurate diagnosis. This challenges conventional diagnostic methods for CD, as they rely on gluten exposure to yield accurate histological and serological findings [85]. The usefulness of novel diagnostic markers, such as gut homing CD8 positive T cells and intraepithelial TCRγδ positive lymphocytes, is highlighted for enabling the detection of CD even in individuals on a GFD [86]. Given the critical importance of early diagnosis and intervention in the context of ART, these advancements decrease the reliance on invasive duodenal biopsy and prolonged gluten challenge procedures. The adoption of these diagnostic tools in clinical settings could enable the detection of previously unrecognized CD cases in patients experiencing repeated ART failure, thereby informing more precise dietary management and enhancing reproductive success [87].

Ultimately, considerable evidence establishes a link between gluten consumption and reproductive health, particularly in CD, where untreated cases are closely related to infertility and adverse pregnancy outcomes. Although further rigorous research is warranted, emerging data indicate that NCGS may play a contributory role in reproductive impairments [82].

The underlying mechanisms likely involve a complex interplay of gluten-induced inflammation, systemic immune dysregulation, nutrient malabsorption critical to reproductive processes, and possible autoimmune effects on hormonal balance, endometrial receptivity, and overall reproductive function [88].

## 9. Conclusions

For individuals experiencing unexplained infertility or recurrent pregnancy loss—especially those with symptoms suggestive of gluten sensitivity or an autoimmune background—screening for CD is an essential step, as supported by several reviewed studies. For individuals with CD, strict adherence to a GFD is crucial and is consistently associated with improved reproductive outcomes.

In individuals with suspected or diagnosed NCGS who present with persistent reproductive abnormalities, a carefully monitored GFD intervention has been proposed in limited studies; however, the current body of evidence remains inconclusive. Given the nutritional limitations associated with GFD, such dietary changes should only be undertaken when medically indicated and under professional supervision. Therefore, while such an approach may be considered for evaluation in select cases, there is currently insufficient data to support its clinical effectiveness in this context.

Maintaining nutritional adequacy and preventing deficiencies, especially during the preconception and gestational periods, requires that dietary interventions, particularly gluten-free diets, be meticulously managed under the guidance of qualified healthcare and nutrition specialists. This review underscores the importance of individualized evidence-based dietary planning, particularly for populations vulnerable to nutritional compromise.

Despite the current knowledge, there remains an urgent need for extensive prospective studies and randomized controlled trials to comprehensively investigate the complex influence of gluten on reproductive health in both individuals with gluten-related disorders and the broader population undergoing ART.

Future studies should aim to identify dependable biomarkers for NCGS to support accurate diagnosis and enable targeted treatment strategies. Furthermore, to ensure optimal outcomes for both mother and fetus, it is essential to develop evidence-based dietary recommendations for pregnant women with celiac disease who adhere to a gluten-free diet. Fertility management in this population may be further enhanced by investigating tailored nutritional therapies that consider individual sensitivities and gut microbiota profiles.

## Figures and Tables

**Table 1:** This table compares the impact of CD on female and male reproductive health. It highlights differences in prevalence, hormonal effects, nutritional deficiencies, and fertility outcomes, along with the potential benefits of a GFD.

Aspect	Female Reproductive Health	Male Reproductive Health
Prevalence of CD in Infertile Individuals	Higher prevalence in women with unexplained infertility (2–8%) [31]	Limited data; underexplored but possible underdiagnosis [22]
Menstrual and Hormonal Effects	Delayed menarche, amenorrhea, early menopause; disrupted ovarian hormone production [21]	Hypogonadism, low testosterone; elevated LH and FSH suggestive of testicular dysfunction [32]
Nutritional Deficiency Impact	Iron, folate, vitamin D, calcium—affects ovulation, endometrial receptivity, and fetal development	Zinc, selenium, folate, B12—impairs spermatogenesis and sperm motility [33]
Endometrial/Testicular Effects	Impaired endometrial receptivity; inflammation and possible autoantibody interference with implantation [16]	Potential autoimmune impact on testicular function; possible anti-sperm antibodies (hypothetical) [34]
Pregnancy/Fertility Outcomes	Increased risk of miscarriage, IUGR, low birth weight, preterm birth [35]	Decreased sperm count, motility, morphology; subfertility [33]
Response to GFD	Restoration of ovulation and menstrual regularity; improved ART outcomes and natural conception [36]	Improved sperm parameters and hormonal balance after GFD adherence [22]
Role of NCGS	Suspected contributor to reproductive symptoms in some women (e.g., infertility, endometriosis-like symptoms) [37]	Largely unknown; minimal data currently available [38]

**Table 2:** This table outlines key mechanisms by which abnormal response to gluten in individuals with CD can affect reproductive health. It describes how immune responses, nutrient malabsorption, and gut-related changes contribute to hormonal imbalance, tissue damage, and impaired fertility.

Mechanism	Description	Effect on Reproductive Health
Immune Dysregulation and Inflammation [52]	Gluten causes immune activation and inflammation that can spread beyond the gut.	Affects oocyte quality, fertilization, implantation, and placenta development.
Nutrient Malabsorption [53]	Damage to the intestine reduces absorption of important vitamins and minerals like iron and folate.	Leads to hormone problems, poor oocyte quality, miscarriage, and birth defects.
Autoimmunity and Molecular Mimicry [5]	Autoantibodies from gluten reaction may attack reproductive tissues by mistake.	Can damage ovaries or placenta and cause fertility issues.
Increased Intestinal Permeability [54]	Gluten can make the gut leak, letting harmful substances enter the bloodstream.	Causes inflammation that may harm reproductive organs.
Gut Microbiota Imbalance [55]	Gluten and gluten-free diets change gut bacteria, affecting immune and hormone balance.	Can disrupt fertility through immune and hormonal changes.
